# *APP^swe^*/*PS1^ΔE9^* mice exhibit low oxygen saturation and alterations of erythrocytes preceding the neuropathology and cognitive deficiency during Alzheimer's disease

**DOI:** 10.1111/cns.14147

**Published:** 2023-03-07

**Authors:** Manli Wang, Xi Chen, Long Niu, Jianli Xu, Hang Yu, Xiaojiao Xu, Qiu Yang, Yang Xiang, Weidong Le

**Affiliations:** ^1^ Institute of Neurology, Sichuan Provincial People's Hospital University of Electronic Science and Technology of China Chengdu China; ^2^ Chinese Academy of Sciences Sichuan Translational Medicine Research Hospital Chengdu China; ^3^ Center for Clinical Research on Neurological Diseases, the First Affiliated Hospital Dalian Medical University Dalian China; ^4^ Liaoning Provincial Key Laboratory for Research on the Pathogenic Mechanisms of Neurological Diseases, the First Affiliated Hospital Dalian Medical University Dalian China

**Keywords:** Alzheimer's disease, oxygen deficiency, oxygen saturation, red blood cells

## Abstract

**Aim:**

The molecular mechanism underlying Alzheimer's disease (AD) pathologies remains unclear. The brain is extremely sensitive to oxygen deprivation, and brief interruptions in oxygen supply may lead to permanent brain damage. The objective here was to access the red blood cell (RBC) physiological alterations and the changes in blood oxygen saturation of an AD model as well as to explore the possible mechanism underlying these pathologies.

**Methods:**

We used female *APP* ^*swe*^ */PS1* ^*ΔE9*^ mice as AD models. Data were collected at the age of 3, 6, and 9 months. In addition to examining classic features of AD, namely cognitive deficiency and Aβ depositions, 24 h blood oxygen saturation was monitored by Plus oximeters in real time. In addition, RBC physiological parameters were measured by blood cell counter using peripheral blood from the epicanthal veins. Furthermore, in the mechanism investigations, the expression of phosphorylated band 3 protein was examined by a series of Western blot analyses, and the levels of soluble Aβ40 and Aβ42 on the membrane of RBCs were determined by ELISA.

**Results:**

Our results showed that the blood oxygen saturation in the AD mice was significantly reduced as early as at 3 months of age, preceding the neuropathological changes and cognitive impairments. Meanwhile, the expression of phosphorylated band 3 protein and levels of soluble Aβ40 and Aβ42 were all elevated in the erythrocytes of the AD mice.

**Conclusion:**

*APP* ^*swe*^ */PS1* ^*ΔE9*^ mice exhibited decreased oxygen saturation together with reduced RBC counts and hemoglobin concentrations at the early stage, which may aid in the development of predictive markers for AD diagnosis. The increased expression of band 3 protein and elevated Aβ40 and Aβ42 levels may contribute to the deformation of RBCs and, in turn, cause the subsequent AD development.

## Graphical abstract

We reveal that the blood oxygen saturation in AD mice is significantly reduced as early as at 3 months of age, preceding the neuropathological changes and cognitive impairments. The results may aid in the development of predictive markers for AD diagnosis.

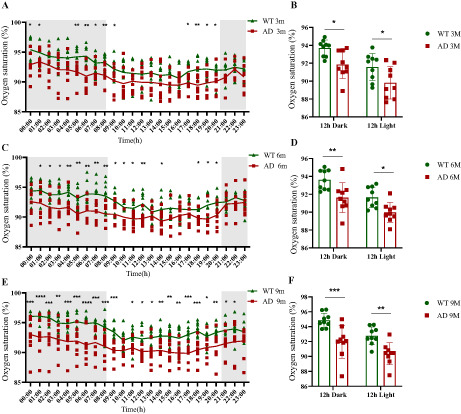

## 1 INTRODUCTION

Alzheimer's disease (AD) is the most common neurodegenerative disease. AD develops slowly in the brain, brain blood vessels, and peripheral tissues and is characterized by age‐associated progressive deterioration of cognitive abilities.^1^, ^2^ More than 90% of AD patients appear to be sporadic and generally with a late age of onset, while familial AD cases usually show symptoms before the age of 65 or even earlier, accounting for 2%–5% of AD cases.^3^ Three genes have been characterized within familial AD cases which are closely related to AD pathogenesis: amyloid precursor protein (APP), presenilin 1 (PSEN1, subunit of γ‐secretase complex), and presenilin 2 (PSEN2, subunit of γ‐secretase complex).^4^ In particular, these three genes exist on the same molecular pathway: the processing of APP by the β‐secretase and γ‐secretase complexes leads to the production of Aβ peptide, a key event in AD pathogeny.^5^ Accordingly, the amyloid cascade hypothesis was proposed, stating that that the mutations on APP or PSEN1/2 are associated with either the increase of Aβ or the elevated ratio of Aβ42 over Aβ40, causing the formation of amyloid plaques and leading to the development of AD.^6^ Certainly, many compelling pieces of evidence support this view, and amyloid plaque accumulation in the brain has been the key subject in disease pathogenesis studies and anti‐amyloid drug developments. However, over the past two decades, the failure of most AD trials suggests that further molecular mechanisms underlying the pathologies are thereby required.^7^, ^8^ The brain needs a constant supply of oxygen and nutrients to function. It is well known that even a brief pause in the flow of oxygen to the brain can lead to chronic brain dysfunction, including irreversible brain damage, decreased cell viability and activity, and a high likelihood of permanent cognitive impairment.^9^ Thus, maintaining normal physiological oxygen concentration is essential for a healthy brain. Erythrocytes are the only cells capable of carrying oxygen and maintaining the aerobic utilization of glucose in tissues.^10^ To date, several remarkable studies have been performed to illustrate the relationship between red blood cell (RBC) metabolic dysfunction and AD.^11^ Accumulation of Aβ in the blood has been reported to impair RBC function and integrity, leading to aggravation of vascular abnormalities.^12^ In addition, RBCs themselves undergo physiological alterations during AD, including disrupted Ca^2+^ permeability, reduced antioxidant enzyme activities, and morphological changes.^13^ Taken together, these RBC pathologies may largely perturb the relevant endogenous metabolic pathways, slow down oxygen release, and cause a brain energy crisis, contributing to AD.^14^ To further explore the alterations in RBCs and blood oxygen saturation during aging as well as the possible underlying mechanism behind these changes, the *APP* ^*swe*^ */PS1* ^*ΔE9*^ mouse model of AD was used in our studies. We found that AD mice developed lower blood oxygen saturation levels, together with decreased hemoglobin concentrations and RBC counts as early as 3 months of age, preceding the onset of Aβ depositions. Further investigations revealed that increased expression of tyrosine phosphorylated band 3 protein and elevated levels of Aβ40 and Aβ42 within the membrane of RBCs may have led to the observed erythrocyte dysfunction by disruption of membrane permeability at the early stage. Taken together, these data suggested that dysfunction of RBC and reduced oxygen saturation in the young AD mice may have promoted the subsequent pathology development.

## 2 MATERIALS AND METHODS

### 2.1 Animals

*APP* ^*swe*^ */PS1* ^*ΔE9*^ mice for breeding were purchased from the Jackson Laboratory (B6C3‐Tg (AβPPswe, PSEN1dE9) 85Dbo/Mmjax, Bar Harbor, MA, USA).^15^ Mice were raised in an environment with good ventilation and sufficient food and water (22 ± 2°C, 12 h/12 h light–dark cycle). In total, 54 mice were divided into six groups, with nine mice in each group. The experimental group was *APP* ^*swe*^ */PS1* ^*ΔE9*^ mice aged 3, 6, and 9 months. Age‐matched wild‐type (WT) mice were used as the control group. All animal procedures were approved by the Institutional Animal Care and Use Committee at Sichuan Provincial People's Hospital. We used female *APP* ^*swe*^ */PS1* ^*ΔE9*^ mice in all experiments.

### 2.2 Morris water maze test

This experiment included a positioning navigation experiment and a space exploration experiment, as previously reported.^16^ The experiment was conducted for a total of 7 days. The first 6 days were training experiments for the mice, and the seventh day was the formal experiment. During training, mice were placed into the pool from each of the four directions, recording the time it took for the mice to find the platform. After the positional navigation experiment was completed, the platform was removed, and each group of mice was placed in the same quadrant of the pool, and the time it took them to first reach the original platform within 60 s, as well as the number of times they crossed the original platform location, were recorded.

### 2.3 Immunofluorescent staining of Aβ plaque in brain tissue

Mice were anesthetized by intraperitoneal injection of pentobarbital sodium (30–90 mg/kg). Mice were perfused with normal saline and 4% paraformaldehyde. Then, 4% paraformaldehyde was added to the brain tissues, and they were stored at 4°C in a refrigerator overnight. The next day, the brain tissue was transferred into 15% and 30% sucrose solution for gradient dehydration until the brain tissue sank to the bottom. The brain tissue was cut with the freezing microtome (Leica). Brain slices with representative layers were selected. After washing with TBST solution, 3% BSA and 0.3% Triton X‐100 were added for blocking. Anti‐β amyloid antibody (Biolegend, 803001, 1:500) was diluted with 3% BSA solution, added to each well, and placed in the 4 °C refrigerator overnight. Goat anti‐mouse IgG (H + L) was conjugated with Alexa Flour 488 (Invitrogen, A‐11001), diluted with 1× PBS solution at 1:500, and incubated for 2 h at 37°C. The brain slices were placed on a glass slide and antifade mounting medium (Solarbio, S2110) was added. High‐magnification images were acquired using confocal laser scanning microscope (20× objective, Olympus, FV3000). Three brain sections from each mouse were imaged and quantified. To conduct the quantitative analysis of Aβ, three optical sections per mouse were processed in ImageJ.

### 2.4 Measurement of blood oxygen saturation

Pulse oximeters (STARR Life Sciences® Corp) were used to monitor the oxygen saturation of experimental mice in real‐time while awake.^17^ The day before the experiment, we shaved the necks of all mice because dark hairs interfere with detecting light signals. At the same time, we clamped the test clips on the necks of the mice and placed them in the experimental cage to adapt to the environment. When the formal experiment began, we turned on the pulse oximeter, selected the awake mode, and set the parameters. The sensor was clipped to the neck of the experimental mouse (where the carotid artery pulsates) and the changes in blood oxygen saturation in the mice were monitored for 24 h. The meeting time was 0:00–24:00.

The instrument recorded oxygen saturation data every 1 second. We averaged the data every 10 min and then used the cosine method to fit these data to obtain the cosine function Y = M + Acos (ωt + φ), where Y is a biological variable that changes with time and A is the amplitude, which is the maximum value of the function fluctuations. After obtaining the function, the Pearson test was used for correlation analysis to determine whether the fitted function differed from the original data. If *p* < 0.05, the variable has circadian rhythms. The 24 h blood oxygen saturation data of each group of mice were expressed as mean ± SEM, and two‐way ANOVA or one‐way ANOVA was used to analyze the data of AD and WT mice of the same age. Graphing and analysis were performed by GraphPad Prism 8 software, and *p* < 0.05 was considered a statistical difference.

### 2.5 Routine blood test

Peripheral blood was taken from the epicanthal veins of mice. After taking the blood, the capillary glass tube was removed, and the mouse's skin was pressed with a cotton ball to stop bleeding. Then, 50 μL of blood was placed in the automatic blood cell counter (PE 6800VET). The system automatically detected various parameters following the manufacturer's instructions.

### 2.6 Sample preparation

Blood samples were centrifuged at 2000 rpm, 4°C for 15 min. Remove the upper serum and the middle layer of leukocytes and platelets. The precipitates were repeatedly dissolved in 40‐fold pre‐chilled Tris–HCl (0.01 moL/L, pH 7.4) for 2 h and centrifuged at 12000 r/min (for mice tissues) or 40,000 *g*/min (for human erythrocyte cultures), 4°C for 20 min until the appearance of white flocculent precipitation.^18^, ^19^ Removed the supernatant and add an appropriate amount of high‐efficiency RIPA buffer (Solarbio, R0010) into the samples, along with a 100:1:1 proportion of phenylmethanesulfonyl fluoride and phosphatase inhibitor. A fraction of lysate was used to perform the protein quantification assay (BCA Protein Assay Kit, Solarbio), and the final protein content of each sample was set to 30 μg for the subsequent Western blot or ELISA analysis.

### 2.7 Western blotting analysis of phosphorylated band 3 protein

30 μg protein for each sample was separated by electrophoresis and transferred to a PVDF membrane that was incubated with phospho‐tyrosine antibody (Cell Signaling, 8954 S, 1:2000) in blocking buffer overnight at 4°C. The membrane was then incubated with goat anti‐rabbit IgG H&L (Abcam, ab97051, 1:4000) in blocking buffer at room temperature for 1 h. Finally, an image was acquired using darkroom development techniques for chemiluminescence.

### 2.8 ELISA analysis of Aβ40 and Aβ42 in RBC membrane

An appropriate amount of RBC membrane sample has been added with RIPA buffer. According to the operating instructions, we determined the levels of Aβ40 and Aβ42 in RBC membrane by using human‐specific ELISA kits (Invitrogen, KHB3481/KHB3544). Finally, we used the microplate reader (Thermo ScientificTM VarioskanTM LUX) to measure the Aβ40 and Aβ42 concentrations of the samples.

### 2.9 Preparation of Aβ_1‐42_ oligomers and human erythrocyte cultures

β‐Amyloid (1–42), human TFA (peptide fragment was synthesized and purified from trifluoroacetate buffer) was purchased from MCE (MCE, HY‐P1363) and was prepared as instructions. Solid Aβ peptide was dissolved in cold hexafluoro‐2‐propanol (HFIP). The peptide was incubated at room temperature for 1 h to establish monomerization and randomization of structure. The HFIP was removed by evaporation, and the resulting peptide was stored as a film at −80°C. The resulting film was all dissolved in anhydrous DMSO to 5 mM and then diluted in PBS buffer to 100 μM with vortexing. Next, the solution was age 48 h at 4–8°C. The sample was then centrifuged at 14000 g for 10 min at 4°C; the soluble oligomers were in the supernatant. When human erythrocytes cultures were prepared, whole blood was collected from 5 informed healthy donors and immediately processed. Plasma separation was obtained by centrifuging at low speed (1000 *g* for 5 min at 4°C), in order to avoid any morphological alteration of erythrocytes induced by mechanical stress. Human erythrocytes were washed three times with a 0.154 M NaCl solution (pH 7.4), in five times their volume. Subsequently, 15 × 10^7^ cells were suspended in 2 mL volume of 1 X TBS in one well of six‐well‐cell culture dishes maintained in a humidified atmosphere with 95% air and 5% CO_2_ for overnight. Then cultures were incubated with 1 μM Aβ_1‐42_ peptide or the same volume 1 X PBS with DMSO, as the control. After 24 h at 37°C, cell suspensions were collected (1000 g for 5 min at 4°C), suspended in 1 X PBS, and used for membrane preparation.^18^

## 3 RESULTS

### 3.1 Cognitive impairments in AD mice

The *APP* ^*swe*^ */PS1* ^*ΔE9*^ mouse model is a classic AD model. We first attempted to confirm the cognitive deficiency already characterized in this model by using Morris water maze tests. The results show that 3‐month‐old AD mice performed equally well as age‐matched WT (Figure S1). However, the 6‐month‐old AD mice exhibited significantly increased escape latencies and decreased platform crossings (Figure S1). Importantly, the variable of escape latency progressively augmented during aging and reached about 40 s in the AD model at the age of 9 months (3.6 times longer than that of 9‐month‐old WT mice). These results demonstrated that, as expected, cognitive impairments appeared in the AD model, and this deficiency was aggravated with aging.

### 3.2 Aβ deposition in the cortex and hippocampus of AD mice

**FIGURE 1:**
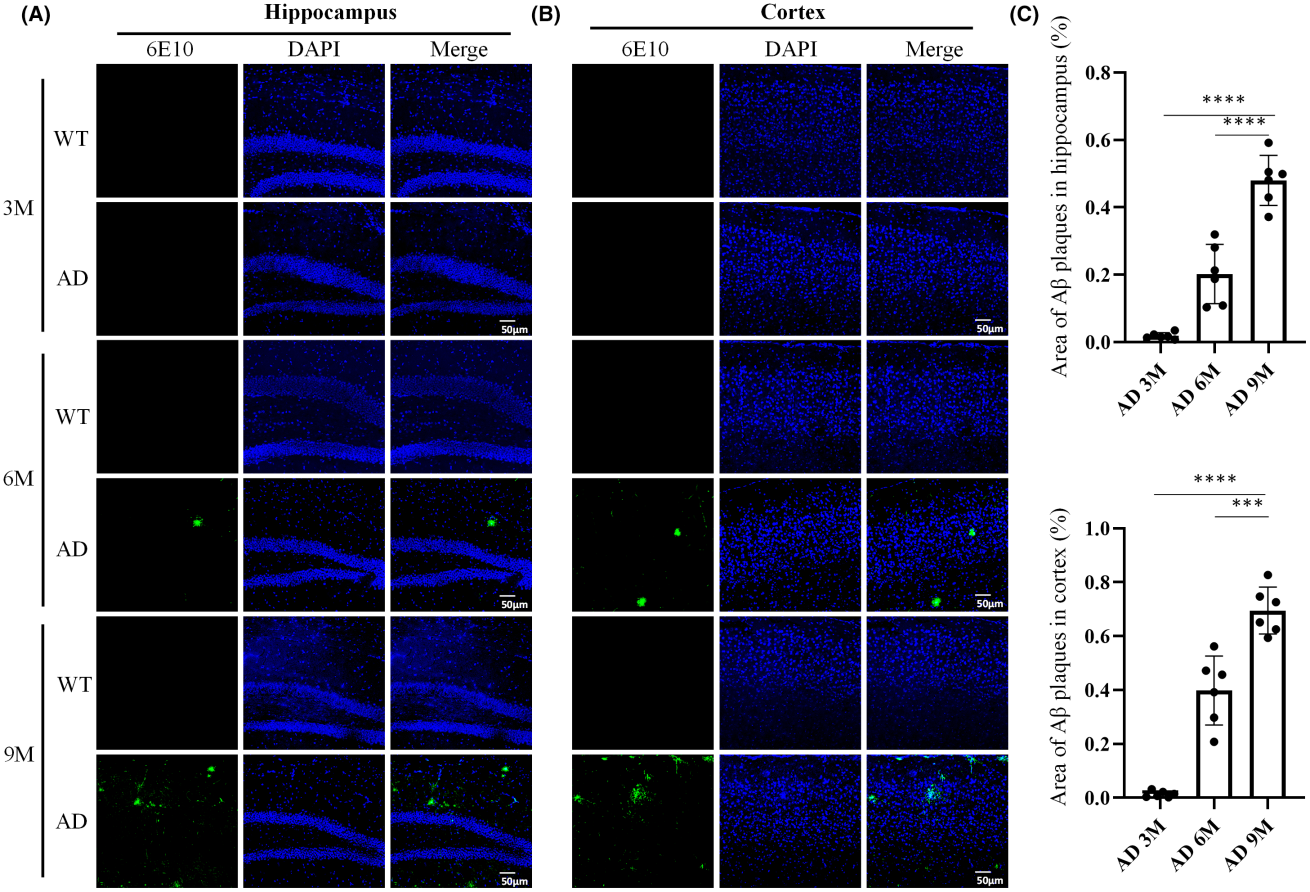
Increased brain Aβ burden in AD mice. (A) Representative images of Aβ plaque in hippocampus in AD and WT mice. Scale bar = 50 μm. (B) Representative images of Aβ plaque in cortex in AD and WT mice. Scale bar = 50 μm. (C) Quantification of Aβ area in hippocampus and cortex of AD and WT mice. *n* = 6 per group, mean ± SEM, one‐way ANOVA, *** *p*< 0.001, **** *p*< 0.0001. Normal distribution was confirmed by Shapiro‐Wilk test (*p*> 0.05).

Beyond the cognitive impairments, another striking feature of this AD model is the occurrence of Aβ plaques, identified primarily in the cortex and hippocampus.^20^ As expected, no Aβ plaque deposition was evident in the cortex and hippocampus of 3‐month‐old AD mice (Figure 1). However, 6‐ and 9‐month‐old AD mice showed marked accumulations of Aβ in the cortex and hippocampus (Figure 1). In particular, the percentage of Aβ plaque area in the cortex and hippocampus of AD mice at 9 months of age was higher than at 6 months of age (Figure 1C). These results reveal that Aβ metabolism was disrupted in the AD model, recapitulating significant AD‐related features.

### 3.3 Altered oxygen saturation in AD mice

**FIGURE 2:**
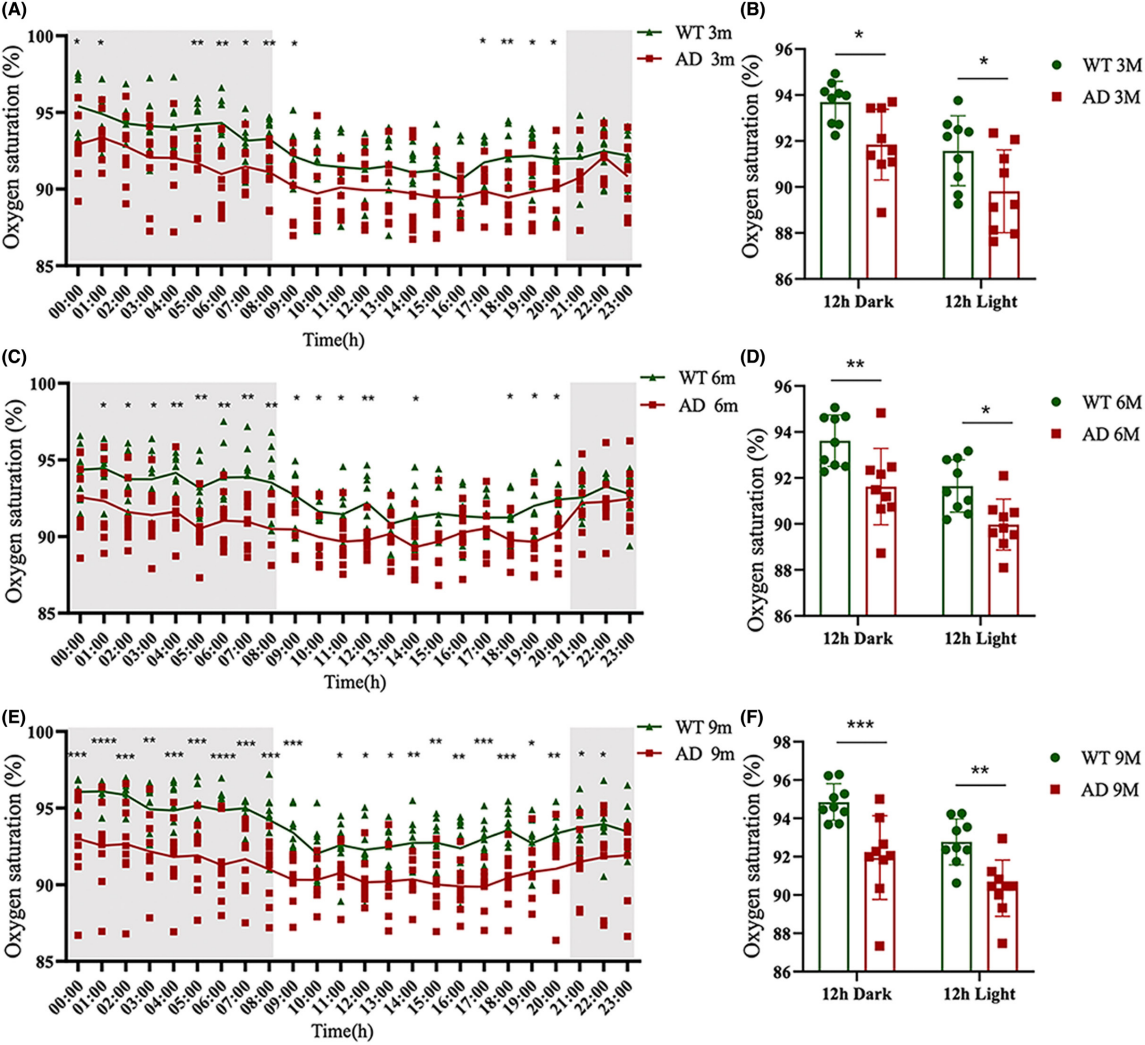
The blood oxygen saturation of AD mice decreased at certain time points. Changes of 24 h blood oxygen saturation in: 3‐month‐old AD mice and WT mice (A); 6‐month‐old AD mice and WT mice (C); and 9‐month‐old AD mice and WT mice (E). *n* = 9 per group, mean ± SEM, multiple t‐test. (D) Quantification of blood oxygen saturation during light period and dark period between: 3‐month‐old AD mice and WT mice (B); 6‐month‐old AD mice and WT mice (D); and 9‐month‐old AD mice and WT mice (F). *n* = 9 per group, mean ± SEM, two‐way ANOVA, * *p* < 0.05, ** *p* < 0.01, *** *p* < 0.001. Normal distribution was confirmed by Shapiro–Wilk test (*p* > 0.05).

Oxygen saturation measures the amount of hemoglobin currently bound to oxygen compared to unbound hemoglobin.^21^ It is an essential indicator to reflect how oxygen is regulated within the body. Since both RBC counts and hemoglobin concentrations were reduced in the AD model, we wondered whether these RBC physiological alterations could affect oxygen saturation and further cause oxygen metabolism deficiency. Therefore, we performed 24 h blood oxygen saturation monitoring in 3‐, 6‐, and 9‐month‐old AD and WT mice using pulse oximetry. As shown in Figure 2, the blood oxygen saturation in AD mice was consistently lower than that in WT mice, potentially due to the reduced RBC counts and hemoglobin concentrations. Interestingly, both WT and AD mice had a higher blood oxygen saturation in the dark period than in the light period, which may be associated with the normal behavior of mice, i.e., mice are usually awake in the dark and sleep in the light.

On the other hand, the time of trough in 24 h blood oxygen saturation in 3‐, 6‐, and 9‐month‐old AD mice were 1 h ahead of age‐matched WT mice (Figure S2), whereas the amplitude of blood oxygen saturation showed no difference between the two genotypes at all stages. These results provide further evidence that oxygen supply in the blood may be disrupted in the AD model.

### 3.4 Decreased red blood cell counts and hemoglobin concentrations in AD mice

The alterations in RBC quantity and hemoglobin concentration have been previously reported in AD patients and AD models.^22^, ^23^ Here, we examined these variables in a longitudinal way (Tables 1 and 2). Our data indicated that the changes in RBC quantity between the two genotypes followed a similar pattern as the changes in hemoglobin concentration (Tables 1 and 2). 3‐, 6‐, and 9‐month‐old AD mice had approximately 20%, 20%, and 23% reduction in RBC counts, respectively, compared to age‐matched WT mice. In addition, 3‐, 6‐, and 9‐month‐old AD mice had approximately 20%, 23%, and 25% lower hemoglobin concentrations, respectively, compared to age‐matched WT mice. Taken together, AD mice developed decreased hemoglobin concentrations and RBC counts as early as 3 months of age, preceding the onset of Aβ depositions.

**TABLE 1:** Comparison of hemoglobin concentration in different groups.

	WT mice	AD mice	*p*
3 M (*n* = 7)	125.43 ± 12.43	100.14 ± 22.07	0.0215
6 M (*n* = 7)	133 ± 4.76	102.43 ± 24.78	0.0076
9 M (*n* = 7)	134.43 ± 11.21	100.86 ± 23.79	0.0055

**TABLE 2:** Comparison of RBC count in different groups.

	WT mice	AD mice	*p*
3 M (*n* = 7)	6.91 ± 0.98	5.54 ± 0.62	0.0088
6 M (*n* = 7)	7.03 ± 0.39	5.62 ± 0.99	0.0043
9 M (*n* = 7)	6.98 ± 0.57	5.34 ± 0.97	0.0023

### 3.5 Increased phosphorylated band 3 protein and higher levels of Aβ40 and Aβ42 in erythrocyte membrane of AD mice

**FIGURE 3:**
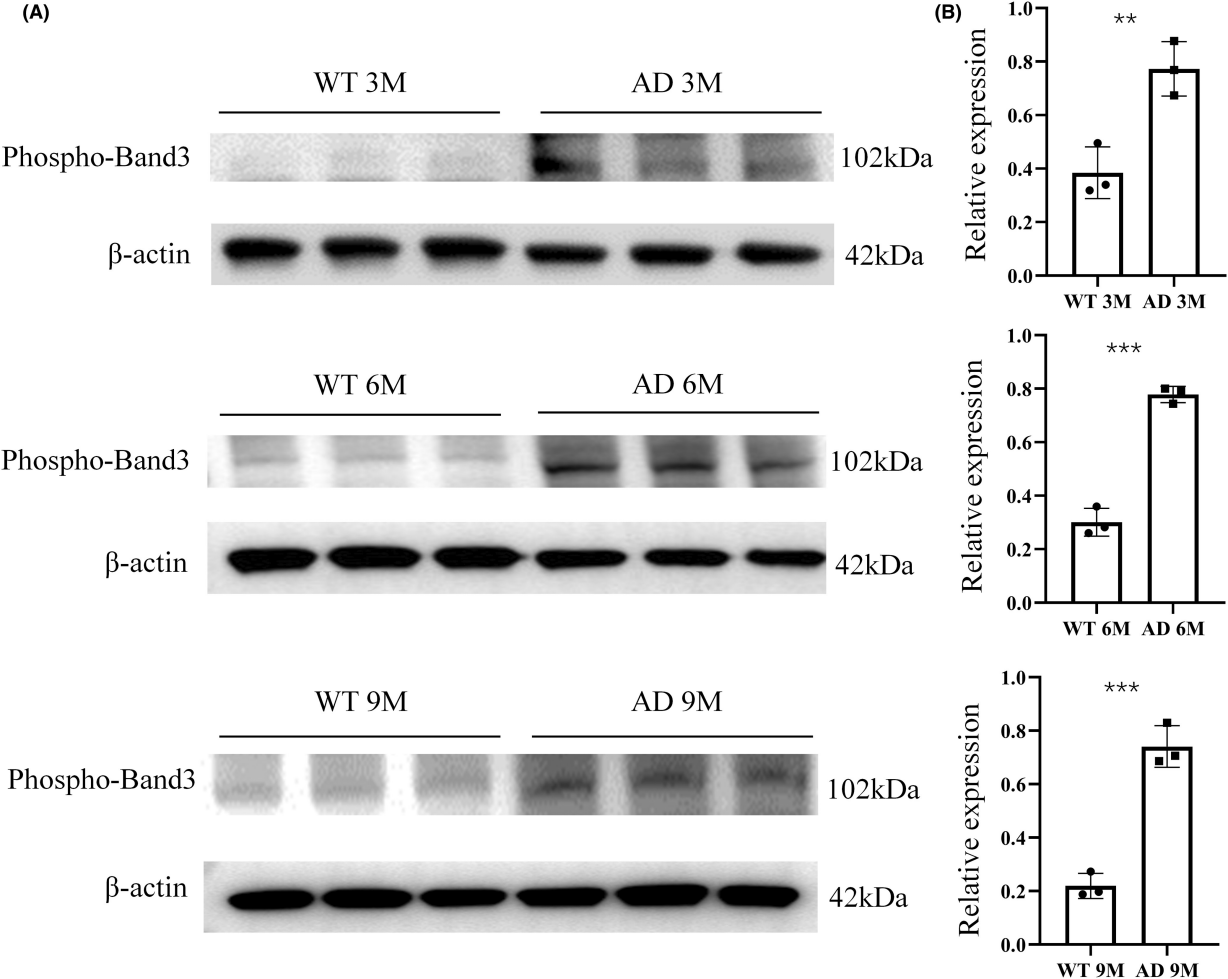
The expression of phosphorylated band 3 protein increased in AD mice. (A) Western blot assays of phosphorylated band 3 protein in the erythrocyte membrane of 3‐, 6‐ and 9‐month‐old AD mice and WT mice. (B) Quantification of band density for phosphorylated band 3 protein in 3‐, 6‐ and 9‐month‐old AD mice and WT mice. *n* = 3 per group, mean ± SEM, t‐test, ** *p* < 0.01, *** *p* < 0.001. Normal distribution was confirmed by Shapiro–Wilk test (*p* > 0.05).

Since significant changes in RBC counts, hemoglobin concentrations, and oxygen saturation were observed in the AD model, we wondered what factors may contribute to these pathological alterations in RBCs. Maintaining the integrity of membrane protein is essential for the normal function of RBC. Band 3 protein is known to comprise about 25% of RBC membrane proteins and is the major substrate for constitutively active protein tyrosine kinase (PTK) and protein tyrosine phosphatase (PTP).^24^ Phosphorylated band 3 protein rises above basal levels upon erythrocyte deoxygenation or cell shrinkage.^25^ Thus, we examined the expressions of phosphorylated band 3 protein on the erythrocyte membrane in the AD model during aging (Figure 3). The results indicated that AD mice had higher phosphorylated band 3 protein levels at all stages, compared to age‐matched WT mice, and this increase was exacerbated with age. To further explore the relationship between phosphorylation levels of band 3 protein and Aβ concentrations, we cultured erythrocytes from 5 control subjects (Table 3) and treated them with oligomeric Aβ_1‐42_ peptide (1 μM). After treatments, the total band 3 proteins were not altered, as shown in Figure S3. However, the phosphorylation of band 3 was increased by 17% in Aβ‐treated erythrocytes membrane samples (Figure S3). Our in vitro data indicated that administration of Aβ_1‐42_ peptide to erythrocyte cultures induces phosphorylation levels of band 3, suggesting that tyrosine phosphorylation‐dependent signaling pathway may impact the peripheral tissues during AD.

**TABLE 3:** Clinical parameters of healthy donors.

	Healthy donors
Subjects	*N* = 5
Age years (mean ± SD)	73.4 ± 3.64
Gender (M/F)	3/2

**FIGURE 4:**
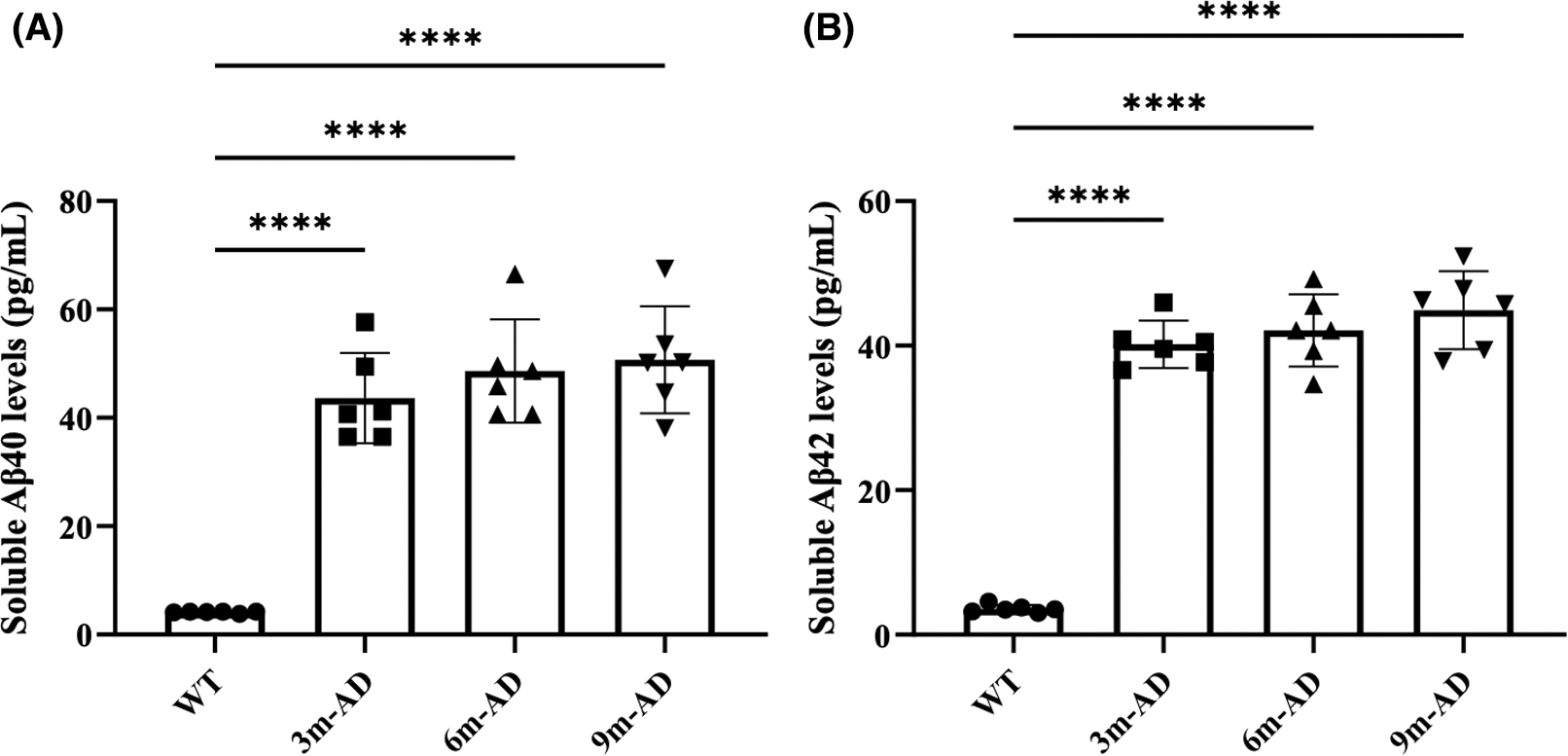
The expression of Aβ40 and Aβ42 on erythrocyte membrane of AD mice increased. Levels of soluble Aβ40 (A) and Aβ42 (B) in AD mice and WT mice. *n* = 6 per group, mean ± SEM, one‐way ANOVA, **** *p* < 0.0001. Normal distribution was confirmed by Shapiro–Wilk test (*p* > 0.05).

Previous studies reported elevated levels of Aβ40 and Aβ42 in the blood and cerebrospinal fluid of AD patients compared to controls.^26^ Therefore, we measured the levels of Aβ40 and Aβ42 in serum and the erythrocyte membrane using ELISA kits (Figures 4 and S4). The data indicated that the levels of Aβ40 and Aβ42 on the erythrocyte membrane of 3‐, 6‐, and 9‐month‐old AD mice significantly increased, compared to age‐matched WT mice. Also, both Aβ40 and Aβ42 levels were significantly increased in the serum of 3‐ and 9‐month‐old AD mice, compared to controls. Taken together, increased expression of phosphorylated band 3 and elevated levels of Aβ40 and Aβ42 may have contributed to the RBC dysfunction and decreased oxygen saturation, ultimately leading to AD development.

## 4 DISCUSSION

The *APP* ^*swe*^ */PS1* ^*ΔE9*^ mice in our studies mirror the significant features known to be associated with AD: progressive cognitive deterioration and Aβ plaque deposition primarily in the cortex and hippocampus. Beyond these classic pathologies, we also found decreased RBC counts and hemoglobin concentrations, as well as lower blood oxygen saturation levels in the AD model. Oxygen saturation is the proportion of total hemoglobin in the blood that is oxyhemoglobin (i.e., hemoglobin bound to oxygen).^14^ As blood flows through tissues, oxygen is released from oxygenated hemoglobin and diffuses into the tissues.^27^ Therefore, a decrease in hemoglobin concentrations and RBC counts may contribute to reducing oxygen saturation. Importantly, when RBC counts, hemoglobin concentrations, and oxygen saturation were all reduced in the AD model, the supply of blood oxygen into the tissues might have been disrupted. The brain is highly sensitive to oxygen flow: even a brief interruption in oxygen supply can lead to permanent brain damage. Therefore, the chronic disrupted oxygen supply in the AD model may contribute to the development of neuronal lesions. In detail, oxygen deficiency is known to affect Aβ metabolism, tau phosphorylation, neuroinflammation, oxidative stress, endoplasmic reticulum stress, and mitochondrial and synaptic dysfunction, thereby promoting neurodegeneration in AD.^28^ Previous studies have suggested that hypoxia is considered as one of the vascular risk factors contributing to the pathogenesis of AD,^28^, ^29^ and hyperbaric oxygen therapy has thereby been subjected to AD patients and AD models in recent years for improving cognitive function. More molecular research revealed that hyperbaric oxygen therapy may alleviate cognitive impairments by mitigating neuroinflammation, decreasing cellular apoptosis, repairing mitochondrial damage, and increasing cerebral blood volume.^30^ Accordingly, enhancing the oxygen saturation by hyperbaric oxygen therapy may be a promising treatment in the future for delaying the progression of the disease.

Our studies suggested that disturbances in oxygen supply may have stimulated Aβ production and Aβ plaque accumulation by activating the expressions of AβPP,^31^ i.e., the early oxygen perturbation may have promoted the subsequent Aβ plaque deposition. The expression of phosphorylated band 3 protein was reported to be upregulated in AD patients and negatively correlated with AD progression.^18^ Increased phosphorylated band 3 alters the cytoskeletal organization by modulating the interactions between band 3 and cytoskeletal anchoring proteins.^32^ We speculate that the elevated phosphorylated band 3 protein disrupted the RBC cytoskeleton, causing deformability of RBCs and affecting the oxygen transport in the blood. On the other hand, Aβ bound to RBCs may cause the accumulation of phospholipid hydroperoxides, thereby inducing oxidative damage to RBCs.^33^ Moreover, since Aβ peptides are normally inserted into the cell membrane, the increased cell membrane permeability of RBCs was characterized during AD.^34^ Our results showed an increase in Aβ40 and Aβ42 on the RBC membrane of the AD model, possibly contributing to the deformability of RBCs and further impaired the oxygen supply.^12^ It has been known for a long time that hypoxia is strongly associated with an increased risk of developing AD.^28^ However, the mechanism behind it is still unclear. In our studies, we revealed that decreased oxygen saturation occurred prior to the Aβ deposition, providing some clues that dysfunctional RBC may contribute to AD pathology by reducing the oxygen capacity in the blood. Furthermore, we found that elevated phosphorylated band 3 proteins were characterized on the membrane of RBC, possibly causing the deformability of cellular structures. On the other hand, the increased amyloid fibrils might be likely attached to erythrocyte membrane and further disrupt the function of RBCs in turn.^23^

In conclusion, we found decreased oxygen saturation together with reduced RBC counts and hemoglobin concentrations at the early stage of the AD model. These results may aid in the development of predictive markers for AD diagnosis. Further mechanism studies suggested that increased expression of band 3 protein and elevated Aβ40 and Aβ42 levels may contribute to the deformability of RBCs and further disruption of the oxygen saturation levels, leading to AD pathology. There is currently no cure for AD, but several treatments may alter disease progression. We expect our studies may provide new insights into AD pathogenesis and therapy.

## AUTHOR CONTRIBUTIONS

LWD designed the experiments. CX contributed to behavior tests. WML and XJL contributed to imaging experiments and data analysis. WML and YH contributed to blood tests and data analysis. WML and XXJ contributed to Western blot tests. CX and YQ contributed to ELISA analysis. NL contributed to the newly added supplementary results, including Figure S4. XJL contributed to the newly added Figure S3. CX, WML, XY, and LWD drafted the manuscript.

## CONFLICT OF INTEREST STATEMENT

The authors declare no conflict of interest, financial or otherwise.

## Supporting information


Figure S1.

Figure S2.

Figure S3.

Figure S4.

## Data Availability

The data that support the findings of this study are available from the corresponding author upon reasonable request.

## References

[1] Scheltens P , Blennow K , Breteler MM , et al. Alzheimer's disease. Lancet. 2016;388(10043):505‐517. 26921134 10.1016/S0140-6736(15)01124-1

[2] Wang X , Wang R , Li J . Influence of sleep disruption on protein accumulation in neurodegenerative diseases. Ageing and Neurodegenerative Diseases. 2022;2(1):4.

[3] Grimm A , Friedland K , Eckert A . Mitochondrial dysfunction: the missing link between aging and sporadic Alzheimer's disease. Biogerontology. 2016;17(2):281‐296. 26468143 10.1007/s10522-015-9618-4

[4] Lanoiselée HM , Nicolas G , Wallon D , et al. APP, PSEN1, and PSEN2 mutations in early‐onset Alzheimer disease: a genetic screening study of familial and sporadic cases. PLoS Med. 2017;14(3):e1002270. 28350801 10.1371/journal.pmed.1002270 PMC5370101

[5] Breijyeh Z , Karaman R . Comprehensive review on Alzheimer's disease: causes and treatment. Molecules. 2020;25(24):5789. 33302541 10.3390/molecules25245789 PMC7764106

[6] Tolar M , Abushakra S , Sabbagh M . The path forward in Alzheimer's disease therapeutics: reevaluating the amyloid cascade hypothesis. Alzheimers Dement. 2020;16(11):1553‐1560. 31706733 10.1016/j.jalz.2019.09.075

[7] Zhang F , Zhong RJ , Cheng C , Li S , Le WD . New therapeutics beyond amyloid‐β and tau for the treatment of Alzheimer's disease. Acta Pharmacol Sin. 2021;42(9):1382‐1389. 33268824 10.1038/s41401-020-00565-5 PMC8379190

[8] Xu Y , Jiang C , Wu J , et al. Ketogenic diet ameliorates cognitive impairment and neuroinflammation in a mouse model of Alzheimer's disease. CNS Neurosci Ther. 2022;28(4):580‐592. 34889516 10.1111/cns.13779 PMC8928920

[9] Burtscher J , Mallet RT , Burtscher M , Millet GP . Hypoxia and brain aging: neurodegeneration or neuroprotection? Ageing Res Rev. 2021;68:101343. 33862277 10.1016/j.arr.2021.101343

[10] Benedik PS , Hamlin SK . The physiologic role of erythrocytes in oxygen delivery and implications for blood storage. Crit Care Nurs Clin North Am. 2014;26(3):325‐335. 25169686 10.1016/j.ccell.2014.04.002

[11] Remigante A , Morabito R , Marino A . Band 3 protein function and oxidative stress in erythrocytes. J Cell Physiol. 2021;236(9):6225‐6234. 33559172 10.1002/jcp.30322

[12] Lan J , Liu J , Zhao Z , et al. The peripheral blood of Aβ binding RBC as a biomarker for diagnosis of Alzheimer's disease. Age Ageing. 2015;44(3):458‐464. 25673873 10.1093/ageing/afv009

[13] Vignini A , Alia S , Pugnaloni S , et al. Erythrocyte membrane fluidity in mild cognitive impairment and Alzheimer's disease patients. Exp Gerontol. 2019;128:110754. 31648010 10.1016/j.exger.2019.110754

[14] Todinova S , Krumova S , Bogdanova D , et al. Red blood Cells' thermodynamic behavior in neurodegenerative pathologies and aging. Biomolecules. 2021;11(10):1500. 34680133 10.3390/biom11101500 PMC8534019

[15] Niu L , Zhang F , Xu X , et al. Chronic sleep deprivation altered the expression of circadian clock genes and aggravated Alzheimer's disease neuropathology. Brain Pathol. 2022;32(3):e13028. 34668266 10.1111/bpa.13028 PMC9048513

[16] Bromley‐Brits K , Deng Y , Song W . Morris water maze test for learning and memory deficits in Alzheimer's disease model mice. J Vis Exp. 2011;53:e2920. 10.3791/2920 PMC3347885 21808223

[17] Inan S , Chen X , Eisenstein EM , et al. Chemokine receptor antagonists enhance morphine's antinociceptive effect but not respiratory depression. Life Sci. 2021;285:120014. 34619167 10.1016/j.lfs.2021.120014

[18] Mallozzi C , Crestini A , D'Amore C , et al. Activation of tyrosine phosphorylation signaling in erythrocytes of patients with Alzheimer's disease. Neuroscience. 2020;433:36‐41. 32156551 10.1016/j.neuroscience.2020.02.050

[19] Liu Z , Chan RB , Cai Z , et al. α‐Synuclein‐containing erythrocytic extracellular vesicles: essential contributors to hyperactivation of monocytes in Parkinson's disease. J Neuroinflammation. 2022;19(1):53. 35193594 10.1186/s12974-022-02413-1 PMC8862590

[20] Zhang F , Zhong R , Li S , et al. Alteration in sleep architecture and electroencephalogram as an early sign of Alzheimer's disease preceding the disease pathology and cognitive decline. Alzheimers Dement. 2019;15(4):590‐597. 30819626 10.1016/j.jalz.2018.12.004

[21] Binene V , Panauwe D , Kauna R , Vince JD , Duke T . Oxygen saturation reference ranges and factors affecting SpO(2) among children living at altitude. Arch Dis Child. 2021;106(12):1160‐1164. 34031027 10.1136/archdischild-2020-321545

[22] Chen SH , Bu XL , Jin WS , et al. Altered peripheral profile of blood cells in Alzheimer disease: a hospital‐based case‐control study. Medicine (Baltimore). 2017;96(21):e6843. 28538375 10.1097/MD.0000000000006843 PMC5457855

[23] Stevenson A , Lopez D , Khoo P , Kalaria RN , Mukaetova‐Ladinska EB . Exploring erythrocytes as blood biomarkers for Alzheimer's disease. J Alzheimers Dis. 2017;60(3):845‐857. 28984593 10.3233/JAD-170363

[24] Pantaleo A , Ferru E , Pau MC , et al. Band 3 erythrocyte membrane protein acts as redox stress sensor leading to its phosphorylation by p (72) Syk. Oxid Med Cell Longev. 2016;2016:6051093. 27034738 10.1155/2016/6051093 PMC4806680

[25] Badior KE , Casey JR . Molecular mechanism for the red blood cell senescence clock. IUBMB Life. 2018;70(1):32‐40. 29240292 10.1002/iub.1703

[26] Olsson B , Lautner R , Andreasson U , et al. CSF and blood biomarkers for the diagnosis of Alzheimer's disease: a systematic review and meta‐analysis. Lancet Neurol. 2016;15(7):673‐684. 27068280 10.1016/S1474-4422(16)00070-3

[27] Fernandes ER , Pires GN , Andersen ML , Tufik S , Rosa DS . Oxygen saturation as a predictor of inflammation in obstructive sleep apnea. Sleep Breath. 2022;26(4):1613‐1620. 34792741 10.1007/s11325-021-02521-x

[28] Zhang F , Niu L , Li S , Le W . Pathological impacts of chronic hypoxia on Alzheimer's disease. ACS Chem Nerosci. 2019;10(2):902‐909. 10.1021/acschemneuro.8b00442 30412668

[29] Zhang F , Zhong R , Qi H , et al. Impacts of acute hypoxia on Alzheimer's disease‐like pathologies in APP(swe)/PS1(dE9) mice and their wild type littermates. Front Neurosci. 2018;12:314. 29867325 10.3389/fnins.2018.00314 PMC5954115

[30] Yang C , Yang Q , Xiang Y , Zeng XR , Xiao J , Le WD . The neuroprotective effects of oxygen therapy in Alzheimer's disease: a narrative review. Neural Regen Res. 2023;18(1):57‐63. 35799509 10.4103/1673-5374.343897 PMC9241400

[31] Li L , Zhang X , Yang D , Luo G , Chen S , Le W . Hypoxia increases Abeta generation by altering beta‐ and gamma‐cleavage of APP. Neurobiol Aging. 2009;30(7):1091‐1098. 18063223 10.1016/j.neurobiolaging.2007.10.011

[32] Condon MR , Feketova E , Machiedo GW , Deitch EA , Spolarics Z . Augmented erythrocyte band‐3 phosphorylation in septic mice. Biochim Biophys Acta. 2007;1772(5):580‐586. 17382523 10.1016/j.bbadis.2007.02.004 PMC1892314

[33] Kiko T , Nakagawa K , Satoh A , et al. Amyloid β levels in human red blood cells. PLoS One. 2012;7(11):e49620. 23166730 10.1371/journal.pone.0049620 PMC3499416

[34] Järemo P , Milovanovic M , Nilsson S , Buller C , Post C , Winblad B . Alzheimer's disease is characterized by more low‐density erythrocytes with increased volume and enhanced β‐amyloid x‐40 content. J Intern Med. 2011;270(5):489‐492. 21486370 10.1111/j.1365-2796.2011.02388.x

