# Supplementary figures and images for: APPswe /PS1ΔE9 mice exhibit low oxygen saturation and alterations of erythrocytes preceding the neuropathology and cognitive deficiency during Alzheimer's disease
Source: CNS Neurosci Ther. 2023 Mar 7;29(7):1889–97. doi: 10.1111/cns.14147 (PMC10546960; doi:10.1111/cns.14147)

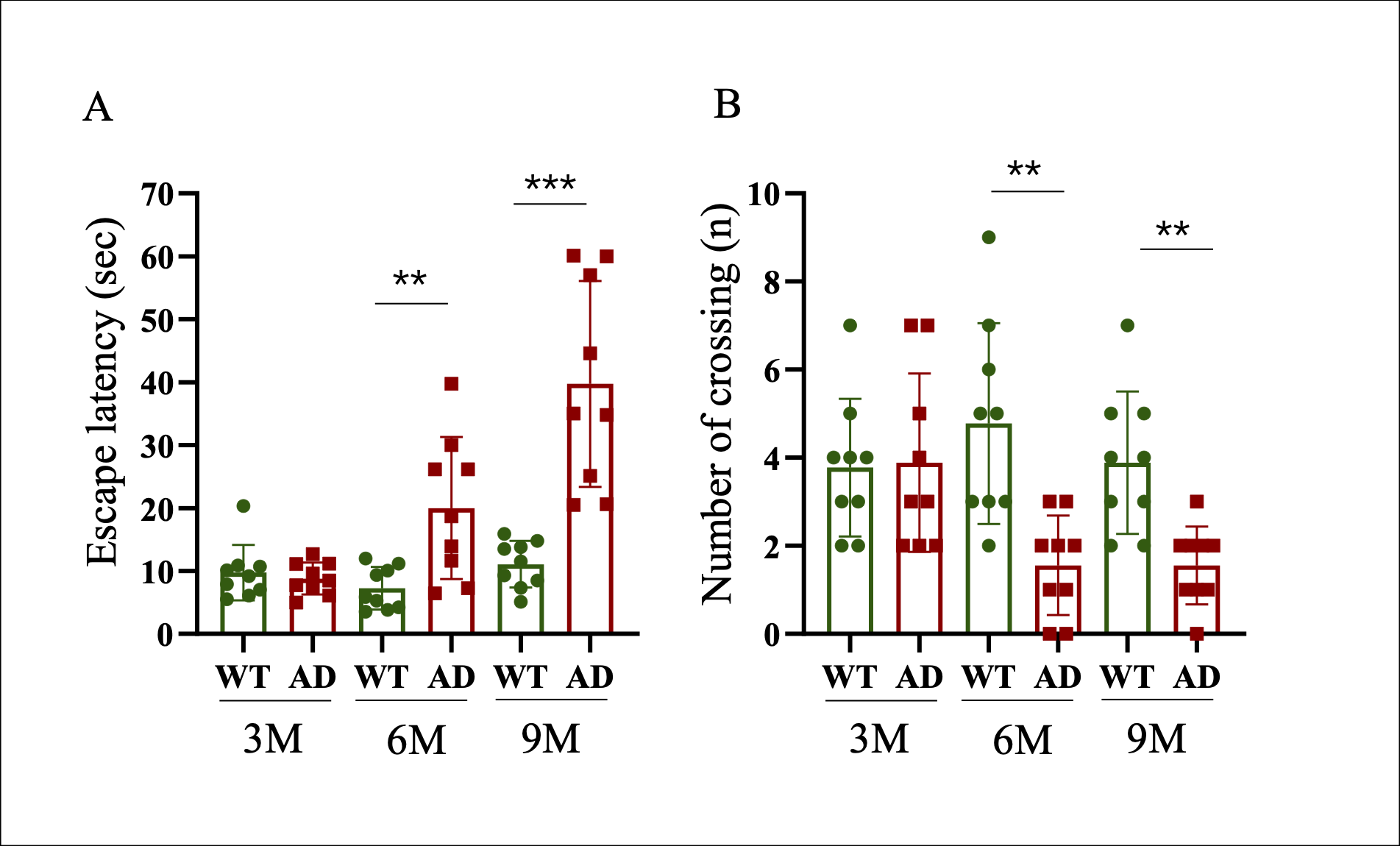

Supplement: Supplementary file 1 — Figure S1. [file CNS-29-1889-s003.tiff]

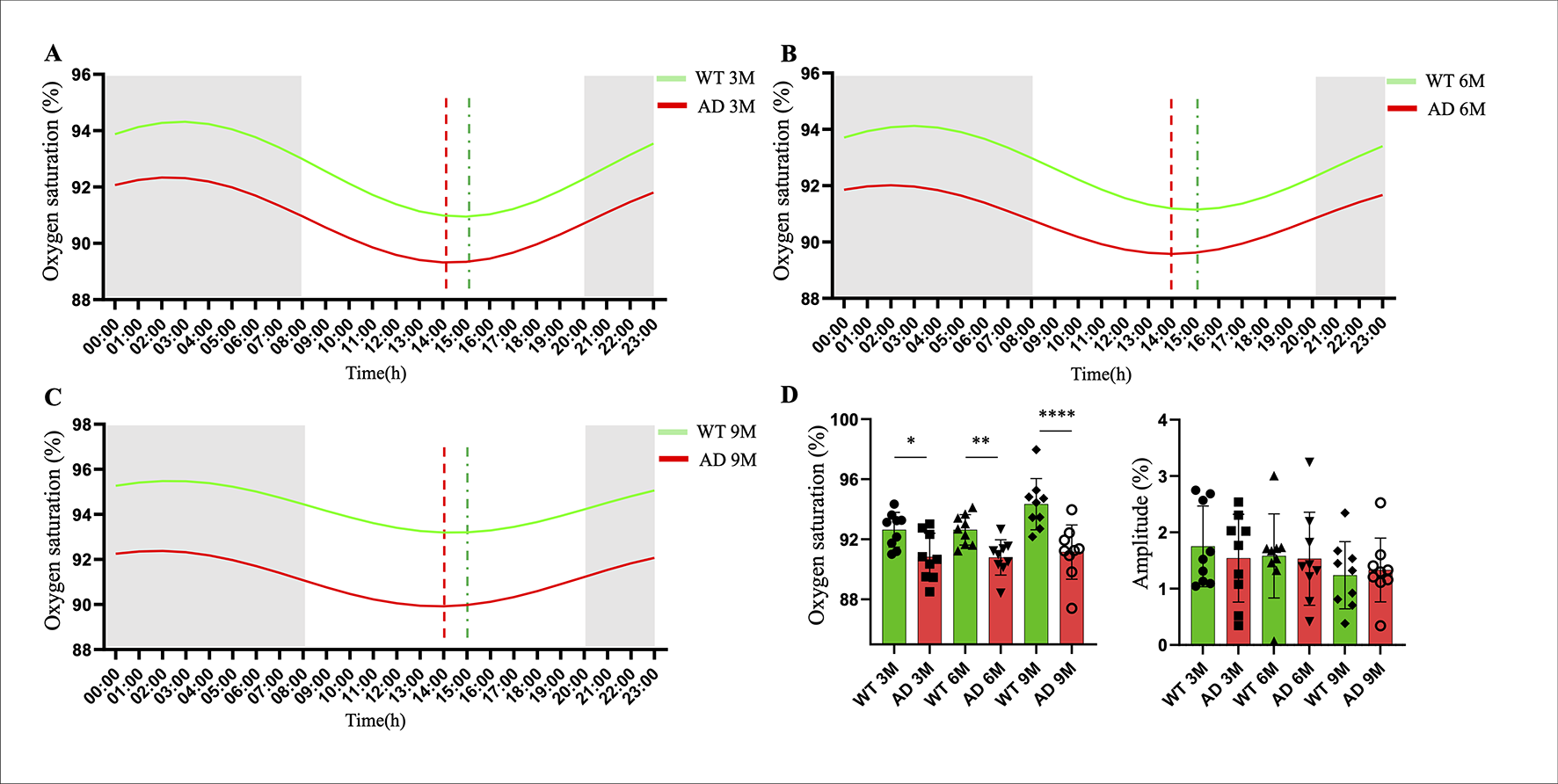

Supplement: Supplementary file 2 — Figure S2. [file CNS-29-1889-s002.tiff]

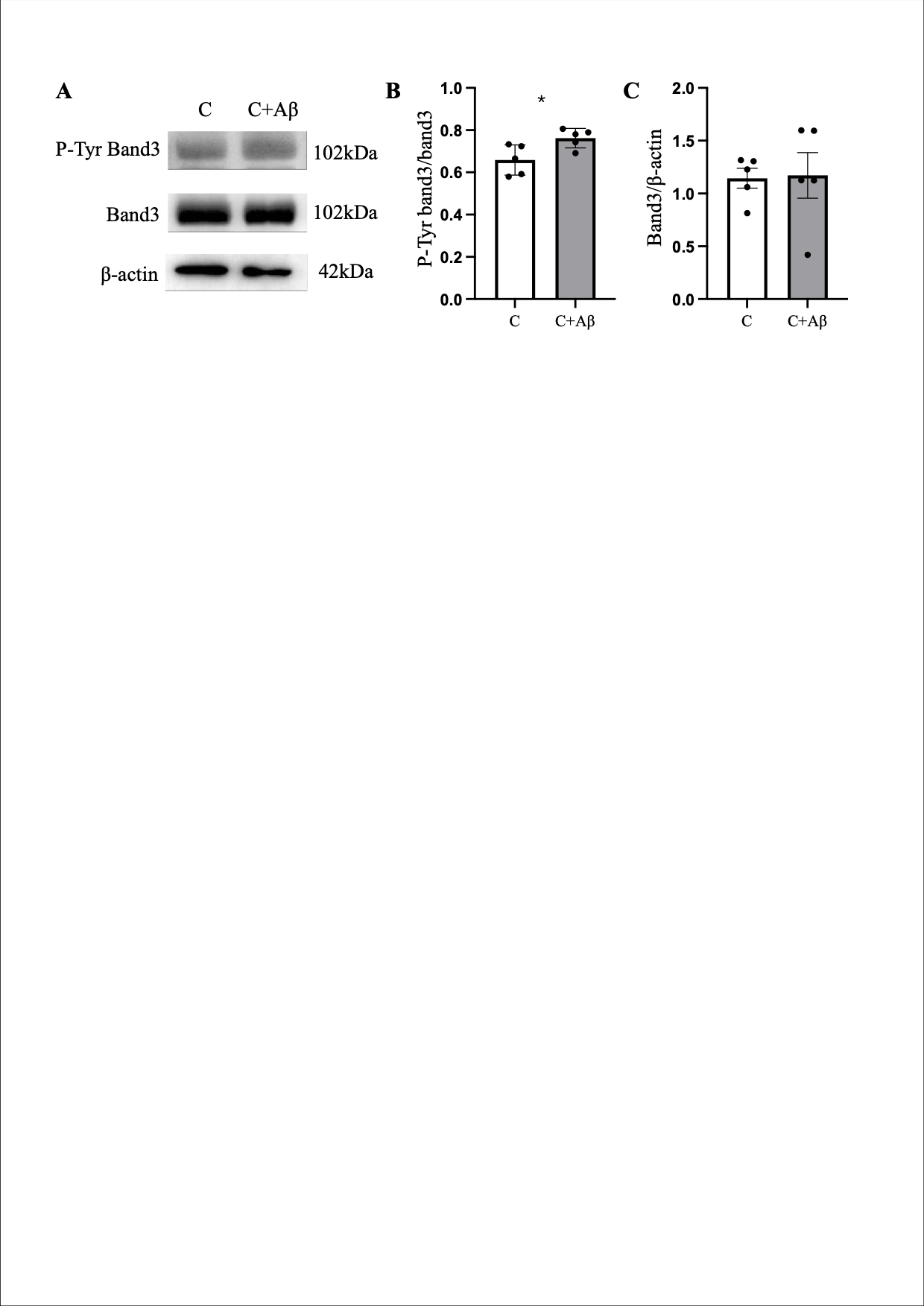

Supplement: Supplementary file 3 — Figure S3. [file CNS-29-1889-s001.tiff]

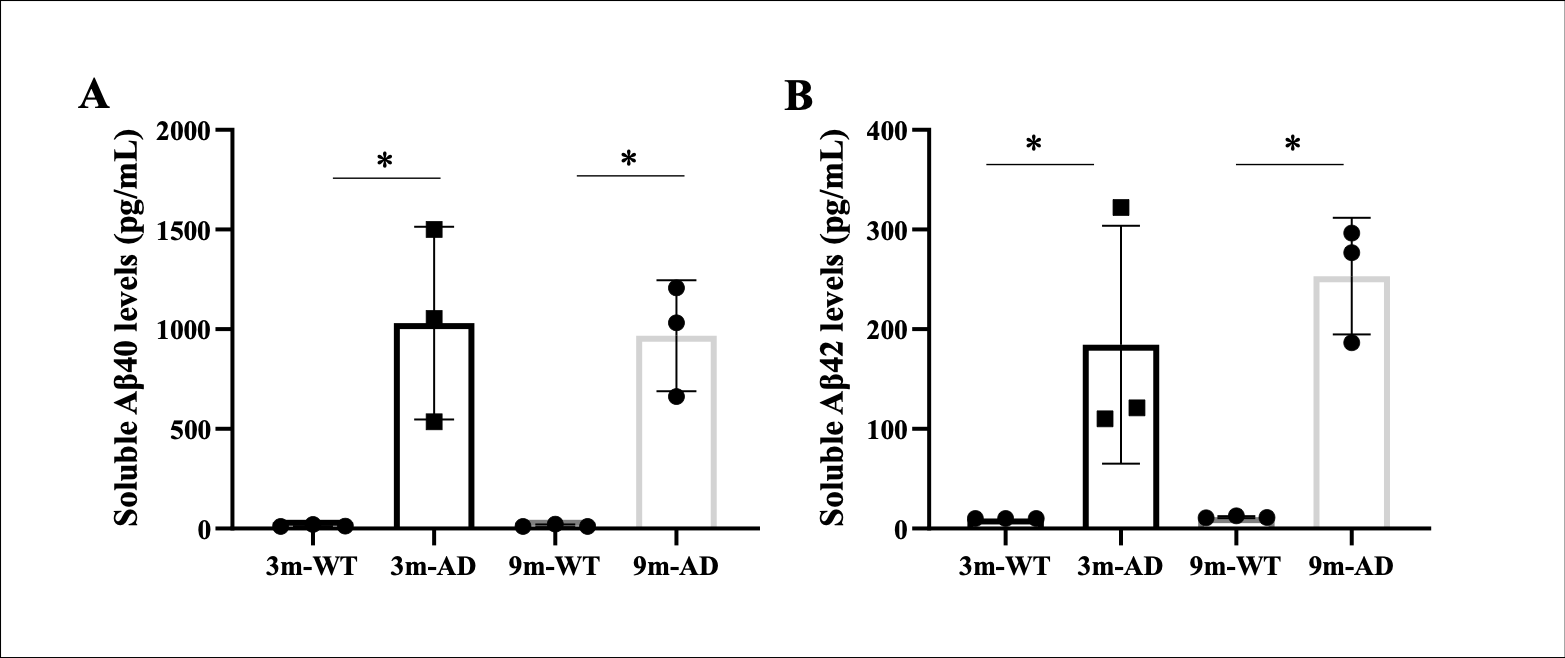

Supplement: Supplementary file 4 — Figure S4. [file CNS-29-1889-s004.tiff]
